# Elevated Anti-Müllerian Hormone Is an Independent Risk Factor for Preterm Birth Among Patients With Overweight Polycystic Ovary Syndrome

**DOI:** 10.3389/fendo.2021.788000

**Published:** 2021-12-08

**Authors:** Mingze Du, Junwei Zhang, Xiaona Yu, Yichun Guan

**Affiliations:** The Reproductive Center, The Third Affiliated Hospital of Zhengzhou University, Zhengzhou, China

**Keywords:** anti-Müllerian hormone, polycystic ovary syndrome, preterm birth, *in vitro* fertilization, body mass index

## Abstract

**Objective:**

To explore whether elevated anti-Müllerian hormone (AMH) levels affect the rate of preterm birth (PTB) among PCOS patients with different BMIs.

**Methods:**

In this retrospective cohort study, patients with PCOS who had undergone IVF/ICSI from January 2017 to December 2019 were included for potential evaluation. A total of 2368 singleton live births from PCOS patients were included. According to the BMI, all the PCOS patients were divided into two groups: BMI<24 kg/m^2^ and BMI≥24 kg/m^2^. In total, 1339 PCOS patients with a BMI<24 kg/m^2^ were grouped according to their serum AMH levels: ① <2.71 ng/ml (n=333), ② 2.71-4.08 ng/ml (n=330), ③ 4.09-6.45 ng/ml (n=351), and ④ >6.45 ng/ml (n=325). Additionally, 1029 cycles of patients with a BMI≥24 kg/m^2^ were grouped according to the serum AMH level: ① <2.71 ng/ml (n=255), ② 2.71-4.08 ng/ml (n=267), ③ 4.09-6.45 ng/ml (n=239), and ④ >6.45 ng/ml (n=268), with <2.71 ng/ml being considered the reference group. The grouping was based mainly on the interquartile range of serum AMH levels. The primary outcome of the study was PTB. The secondary outcomes were low birth weight (LBW), small for gestational age (SGA), macrosomia and large for gestational age (LGA).

**Results:**

Regarding PCOS patients with a BMI<24 kg/m^2^, compared with the PTB rate of the AMH <2.71 ng/ml group, the PTB rates of the different groups were not significantly different (AMH 2.71-4.08, AOR (95% CI)=1.01 (0.52-2.00), P=0.99; AMH 4.09-6.45, AOR (95% CI)=0.93 (0.45-1.91), P=0.85; AMH>6.45, AOR (95% CI)=0.78 (0.35-1.73), P=0.54). Regarding PCOS patients with a BMI ≥24 kg/m^2^, compared with the PTB rate of the AMH <2.71 ng/ml group, the PTB rate of the AMH>6.45 ng/ml group was significantly higher (OR=2.47; 95% CI=1.34-4.55). After multiple logistic regression analysis, the risk of PTB in the AMH>6.45 ng/ml group was 2.1 times that in the AMH<2.71 ng/ml group (AOR=2.1, 95% CI=1.01-4.37, P=0.04). However, no statistically significant difference was found in the rate of SGA, LBW, macrosomia or LGA among patients in the different serum AMH groups.

**Conclusion:**

For PCOS patients, a BMI≥24 kg/m^2^ plus serum AMH>6.45 ng/ml (75th percentile) is an independent risk factor for PTB.

## Introduction

Polycystic ovary syndrome (PCOS) is characterized by ovarian dysfunction and other cardinal features—namely, hyperandrogenism and polycystic ovary (PCO) morphology. Additionally, menstrual irregularities, signs of androgen excess, obesity and infertility are common clinical manifestations (1). The clinical features of PCOS patients are heterogeneous, and the most commonly used diagnostic criteria are the Rotterdam criteria, established in 2003 (1). PCOS occurs primarily in women of reproductive age. The prevalence of PCOS has been reported to range from 8% to 13% worldwide (2–4), and the prevalence of PCOS among Chinese women aged 19-45 years is close to 5.6% (5). The rapid development of assisted reproductive technology (ART) made this method crucial for the treatment of infertility, and it is widely used in the PCOS population compared with the non-PCOS population (6). The safety of ART for the offspring of the PCOS population has been a focus of research. Current studies have reported that PCOS patients have increased rates of pregnancy complications, including pregnancy-induced hypertension and gestational diabetes mellitus, and an increased miscarriage rate (7). Studies have also indicated that the preterm birth (PTB) rate of PCOS patients is increased, regardless of whether pregnancy is achieved spontaneously or through IVF (8–10).

Anti-Müllerian hormone (AMH) is a dimeric glycoprotein and a transforming growth factor-beta superfamily member that acts on tissue growth and differentiation (11). AMH is produced by granulosa cells from preantral and antral follicles, and its expression is restricted in growing follicles until they have reached the size and differentiation state at which they are selected for dominance *via* pituitary FSH (12). The AMH level basically reflects the follicular reserve in the ovary.

Compared with women without PCOS, those with PCOS have more antral follicles (13, 14). Thus, the circulating AMH levels in women with PCOS are two to three times higher than those in healthy controls (13, 15), a finding that may be related not only to excessive accumulation of antral follicles but also to increased granulosa cell AMH secretion (16–18). Therefore, the serum AMH level may be related to the severity of PCOS, and whether a high AMH level influences offspring remains uncertain. To our best knowledge, only 2 clinical studies have analyzed the relationship between elevated AMH levels and premature delivery of offspring after IVF (9, 10). However, the data are limited, and current studies do not distinguish the effects of different body mass indices (BMIs). Even so, the BMI is related to PCOS severity and offspring outcomes (19–21). Therefore, the impact of AMH levels on the offspring of PCOS patients in different BMI groups must be further analyzed. This study aimed to evaluate whether elevated AMH levels affect the rate of preterm birth (PTB) among PCOS patients with different BMIs.

## Materials and Methods

### Study Design and Population

This was a single-center retrospective cohort study approved by the Ethics Committee of the Third Affiliated Hospital of Zhengzhou University. Patients with PCOS who had undergone *in vitro* fertilization (IVF)/intracytoplasmic sperm injection (ICSI) at the Reproductive Center of the Third Affiliated Hospital of Zhengzhou University from January 2017 to December 2019 were included for potential evaluation in this study. The diagnosis of PCOS was based on the Rotterdam criteria established in the 2003 Rotterdam consensus workshop, which required that at least two of the following three criteria were met: oligomenorrhea and/or anovulation, clinical and/or biochemical signs of hyperandrogenism, and polycystic ovaries on ultrasound scanning (1). Only cycles with fresh embryo transfer and singleton delivery were included. Cycles with pregnancy-induced hypertension, gestational diabetes mellitus, adenomyosis, uterine malformations, endometrial polyps and preimplantation genetic testing (PGT) were excluded. The baseline and cycle information of all the patients was obtained from our electronic medical record system, and the information on newborns was obtained from the follow-up records of professional nurses.

According to the BMI, PCOS patients were divided into two groups: BMI<24 kg/m^2^ and BMI≥24 kg/m^2^. Next, we divided the cycles into four groups based on the AMH levels: ① <2.71 ng/ml, ② 2.71-4.08 ng/ml, ③ 4.09-6.45 ng/ml, and ④ >6.45 ng/ml. The <2.71 ng/ml group was considered the reference group, and grouping was based primarily on the interquartile range of the serum AMH levels.

### Clinical Protocols

The mothers were subjected to our center’s conventional ovulation stimulation protocols—namely, gonadotrophin-releasing hormone (GnRH) agonist protocols or flexible GnRH antagonist protocols. The specific ovulation protocol details have been described in our previous studies (22, 23). For both protocols, the dose of follicle-stimulating hormone (rFSH) was adjusted according to the follicle response, as determined by transvaginal ultrasound monitoring and blood hormone level measurements. When the diameter of the dominant follicle was greater than 20 mm or when at least three follicles reached 18 mm in diameter, ovulation induction was triggered with 5000 to 10000 IU human chorionic gonadotrophin (hCG; Lizhu Pharmaceutical Trading, China). Oocyte retrieval was performed 36 hours later. Routine IVF or ICSI was performed according to the man’s semen quality. Women underwent the transfer of 1 to 2 cleavage-stage embryos on the third day or transfer of a single fresh blastocyst on the fifth day after fertilization. Routine corpus luteum support—namely, oral dydrogesterone (10 mg twice daily) (Abbott Co. America)—was initially provided on the day of oocyte retrieval, and intravaginal administration of 90 mg of a progesterone sustained-release vaginal gel (Merck Co. Germany) was performed. Corpus luteum support was performed until at least 55 days after transplantation if pregnancy occurred. The serum hormone levels were tested using the Cobas C8000 system (Roche Diagnostics, Germany), and the measurement approaches were described in our previous study (22).

### Outcome Measures and Definition

PTB was the main outcome for this analysis and was defined as a birth occurring after 28 weeks and before 37 completed weeks of gestation. The secondary outcome measures were a low birth weight (LBW, defined as a neonatal birth weight less than 2500 g), small for gestational age [SGA, defined as a birth weight less than the 10th percentile for gestational age, per the weight criteria for Chinese newborns (24)], macrosomia (defined as a neonatal birth weight greater than 4000 g) and large for gestational age [LGA, defined as a birth weight larger than the 90th percentile for the sex-specific birth weight, per the sex-specific birth weight criteria for weight among Chinese newborns (24)].

### Statistical Analysis

All statistical management and analyses were performed using SPSS software, version 22.0.

The one-sample Kolmogorov–Smirnov test was used to check for the normality of continuous variables. Continuous variables with abnormal distributions were expressed as the median (P25, P75), and the between-group differences were assessed using the Wilcoxon rank-sum test. Categorical variables were represented as the number of cases (n) and percentage (%). The means from chi-squared analyses were used to assess the differences between groups using Fisher’s exact test when necessary. For the outcome measures—namely, PTB, LBW, SGA, macrosomia and LGA—multiple logistic regression was used to adjust for the baseline characteristics. Odds ratios (ORs) and adjusted odds ratios (AORs) with 95% confidence intervals (CIs) were calculated. P<0.05 was considered to be statistically significant.

## Results

### Study Population

In this study, 2368 singleton live births from PCOS patients were included in the analysis from January 2017 to December 2019.

In total, 1339 PCOS patients with a BMI<24 kg/m^2^ were grouped according to the serum AMH level: ① <2.71 ng/ml (n=333), ② 2.71-4.08 ng/ml (n=330), ③ 4.09-6.45 ng/ml (n=351), and ④ >6.45 ng/ml (n=325).

A total of 1029 cycles were evaluated from patients with a BMI≥24 kg/m^2^, and they were grouped according to the serum AMH level: ① <2.71 ng/ml (n=255), ② 2.71-4.08 ng/ml (n=267), ③ 4.09-6.45 ng/ml (n=239), and ④ >6.45 ng/ml (n=268).

### Baseline and Cycle Characteristics

Detailed information on the baseline and cycle characteristics between groups is presented in **Table 1**. Significant differences were found in the maternal age (P<0.001), paternal age (P<0.001), duration of infertility (P<0.001), type of infertility (P<0.001), basal serum FSH level (P<0.001), basal antral follicle count (P<0.001), dosage of gonadotropin (P<0.001), duration of ovarian stimulation (P<0.001), number of oocytes retrieved (P=0.01), endometrial thickness on the trigger day (P=0.01) and fertilization method (P=0.045) between the BMI<24 kg/m^2^ group and BMI≥24 kg/m^2^ group. The neonatal birth weight was higher in the BMI≥24 kg/m^2^ group than in the BMI<24 kg/m^2^ group (3500.0 (3150.0, 3800.0) vs. 3300.0 (3050.0, 3600.0); P<0.001). Differences were found in the gestational week at delivery between the groups (P=0.01). The serum AMH level (P=0.95), number of 2PN (P=0.45), number of available embryos (P=0.74), stage of embryo transfer (P=0.57), number of embryos transferred (P=0.97) and sex of the newborn (P=0.62) were comparable between the groups. The incidence of OHSS was higher in the BMI<24 kg/m^2^ group than in the BMI≥24 kg/m^2^ group (6.2% vs. 3.1%; P=0.001).

**Table 1:** Baseline and cycle characteristics among patients with PCOS.

Parameter	BMI<24 kg/m^2^ (N = 1339)	BMI≥24 kg/m^2^ (N = 1029)	*P*-value
Maternal age (years)	29.0 (27.0, 32.0)	30.0 (27.0, 33.0)	<0.001
Paternal age (years)	30.0 (28.0, 33.0)	31.0 (28.0, 34.0)	<0.001
Duration of infertility (years)	3.0 (2.0, 4.0)	3.0 (2.0, 5.0)	<0.001
Type of infertility (%)			<0.001
Primary infertility	55.3 (741/1339)	45.2 (465/1029)	
Secondary infertility	44.7 (598/1339)	54.8 (564/1029)	
Basal serum FSH level (IU/L)	6.7 (5.6, 7.8)	6.4 (5.4, 7.5)	<0.001
AMH (ng/ml)	4.1 (2.7, 6.4)	4.0 (2.7, 6.6)	0.95
Basal antral follicle count	19 (15, 24)	21 (15, 24)	<0.001
Dosage of gonadotropins (IU)	1950.0 (1500.0, 2625.0)	2562.0 (1950.0, 3300.0)	<0.001
Duration of ovarian stimulation (days)	13 (12, 14)	13 (12, 15)	<0.001
No. of oocytes retrieved	15 (12, 19)	15 (11, 18)	0.01
No. of 2PN	10 (7, 13)	10 (6, 13)	0.45
No. of available embryos	8 (5, 11)	8 (5, 11)	0.74
Endometrial thickness on the trigger day (mm)	11.0 (10.0, 13.0)	11.7 (10.0, 13.0)	0.01
OHSS rate (%)	6.2 (83/1339)	3.1 (32/1029)	0.001
Fertilization method (%)			0.045
IVF	73.0 (977/1339)	76.6 (788/1029)	
ICSI	27.0 (362/1339)	23.4 (241/1029)	
Stage of embryo transfer (%)			0.57
Cleavage embryo	50.3 (674/1339)	51.5 (530/1029)	
Blastocyst	49.7 (665/1339)	48.5 (499/1029)	
No. of embryo transfers (%)			0.97
1	54.9 (735/1339)	54.8 (564/1029)	
2	45.1 (604/1339)	45.2 (465/1029)	
Neonatal birth weight (g)	3300.0 (3050.0, 3600.0)	3500.0 (3150.0, 3800.0)	<0.001
Gestational weeks at delivery (weeks)	39 (38, 40)	39 (38, 40)	0.01
Gender of newborn (%)			0.62
Male	56.6 (758/1339)	55.6 (572/1029)	
Female	43.4 (581/1339)	44.4 (457/1029)	

The data are presented as medians (P25, P75) for continuous variables and %(n/N) for categorical variables.

### Neonatal and Perinatal Outcomes

To adjust for the influence of possible confounding factors, we conducted multiple logistic regression analysis. The factors included in the regression model included the maternal age (continuous variable), BMI (continuous variable), duration of infertility (continuous variable), type of infertility (primary/secondary infertility), basal antral follicle count (continuous variable), fertilization method (IVF/ICSI), endometrial thickness on the trigger day (continuous variable), developmental stage of the embryo (cleavage/blastocyst) and serum AMH level (<2.71 (reference)/2.71-4.08/4.09-6.45/>6.45 ng/ml).

Regarding PCOS patients with a BMI<24 kg/m^2^, compared with the PTB rate of the AMH <2.71 ng/ml group, those of the different groups were not significantly different (AMH 2.71-4.08, AOR (95% CI)=1.01 (0.52-2.00), P=0.99; AMH 4.09-6.45, AOR (95% CI)=0.93 (0.45-1.91), P=0.85; AMH>6.45, AOR (95% CI)=0.78 (0.35-1.73), P=0.54). The different degrees of serum AMH elevation had no significant effect on SGA, LBW, macrophages, or LGA. The data are presented in **Table 2**.

**Table 2:** Neonatal outcomes among patients with PCOS and a BMI<24 kg/m^2^ among the groups.

	Serum AMH level (ng/ml)			
	<2.71 (N = 333)	2.71-4.08 (N = 330)	4.09-6.45 (N = 351)	>6.45 (N = 325)
**SGA**				
% (n/N)	5.4 (18/333)	4.2 (14/330)	7.1 (25/351)	6.2 (20/325)
OR (95%CI)	1	0.78 (0.38-1.59)	1.34 (0.72-2.51)	1.15 (0.60-2.21)
Adjusted OR (95% CI)	1	0.71 (0.34-1.50)	1.17 (0.58-2.37)	1.02 (0.47-2.21)
Adjusted P value		0.37	0.70	0.96
**LBW**				
% (n/N)	4.2 (14/333)	5.2 (17/330)	4.8 (17/351)	5.2 (17/325)
OR (95% CI)	1	1.24 (0.60-2.55)	1.16 (0.56-2.39)	1.26 (0.61-2.60)
Adjusted OR (95% CI)	1	1.15 (0.54-2.47)	0.99 (0.44-2.22)	0.95 (0.40-2.25)
Adjusted P value	–	0.72	0.97	0.91
**Macrosomia**				
% (n/N)	7.2 (24/333)	8.5 (28/330)	8.3 (29/351)	9.2 (30/325)
OR (95% CI)	1	1.19 (0.68-2.11)	1.16 (0.66-2.04)	1.31 (0.75-2.29)
Adjusted OR (95% CI)	1	1.09 (0.60-1.98)	1.06 (0.56-1.99)	1.04 (0.53-2.03)
Adjusted P value	–	0.78	0.86	0.92
**LGA**				
% (n/N)	14.1 (47/333)	17.0 (56/330)	13.7 (48/351)	14.2 (46/325)
OR (95% CI)	1	1.24 (0.82-1.90)	0.96 (0.63-1.49)	1.00 (0.65-1.56)
Adjusted OR (95% CI)	1	1.19 (0.77-1.86)	0.95 (0.58-1.54)	0.90 (0.53-1.52)
Adjusted P value	–	0.44	0.83	0.69
**PTB**				
% (n/N)	5.4 (18/333)	5.8 (19/330)	6.3 (22/351)	5.8 (19/325)
OR (95% CI)	1	1.07 (0.55-2.08)	1.17 (0.62-2.22)	1.09 (0.56-2.11)
Adjusted OR (95% CI)	1	1.00 (0.52-2.00)	0.93 (0.45-1.91)	0.78 (0.35-1.73)
Adjusted P value	–	0.99	0.85	0.54

The analyses were adjusted for the maternal age, BMI, duration of infertility, type of infertility (Primary/Secondary infertility), basal antral follicle count, fertilization method (IVF/ICSI), endometrial thickness on the trigger day and development stage of the embryo (cleavage/blastocyst). OR, odds ratio; CI ,confidence interval; SGA ,small-for-gestational age; LBW ,low birthweight; LGA, large-for-gestational age; PTB, preterm birth.

Regarding PCOS patients with a BMI≥24 kg/m^2^, compared with the PTB rate of the AMH <2.71 ng/ml group, that of the AMH>6.45 ng/ml group was significantly higher (OR=2.47; 95% CI=1.34-4.55). After multiple logistic regression analysis, the risk of PTB in the AMH>6.45 ng/ml group was 2.1 times that in the AMH<2.71 ng/ml group (AOR=2.1; 95% CI=1.01-4.37; P=0.04). However, no statistically significant difference was found in the rate of SGA, LBW, macrosomia, or LGA among the groups of patients with different serum AMH levels. The specific data are presented in **Table 3**.

**Table 3:** Neonatal outcomes among patients with PCOS and a BMI ≥24 kg/m^2^ among the groups.

	Serum AMH level (ng/ml)			
	<2.71 (N = 255)	2.71-4.08 (N = 267)	4.09-6.45 (N = 239)	>6.45 (N = 268)
**SGA**				
% (n/N)	3.9 (10/255)	4.1 (11/267)	4.6 (11/239)	4.9 (13/268)
OR (95% CI)	1	1.05 (0.44-2.52)	1.18 (0.49-2.84)	1.25 (0.54-2.90)
Adjusted OR (95% CI)	1	0.98 (0.39-2.44)	1.04 (0.39-2.76)	1.13 (0.41-3.15)
Adjusted P value		0.97	0.94	0.82
**LBW**				
% (n/N)	5.9 (15/255)	4.1 (11/267)	4.6 (11/239)	4.1 (11/268)
OR (95% CI)	1	0.69 (0.31-1.53)	0.77 (0.35-1.72)	0.69 (0.31-1.52)
Adjusted OR (95% CI)	1	0.64 (0.28-1.47)	0.63 (0.26-1.56)	0.51 (0.19-1.36)
Adjusted P value		0.29	0.32	0.18
**Macrosomia**				
% (n/N)	14.9 (38/255)	14.6 (39/267)	13.4 (32/239)	18.7 (50/268)
OR (95% CI)	1	0.98 (0.60-1.59)	0.88 (0.53-1.47)	1.31 (0.83-2.08)
Adjusted OR (95% CI)	1	0.89 (0.53-1.47)	0.71 (0.40-1.25)	0.99 (0.56-1.74)
Adjusted P value		0.63	0.23	0.96
**LGA**				
% (n/N)	27.1 (69/255)	25.1 (67/267)	24.3 (58/239)	32.5 (87/268)
OR (95% CI)	1	0.90 (0.61-1.34)	0.86 (0.58-1.30)	1.30 (0.89-1.89)
Adjusted OR (95% CI)	1	0.82 (0.54-1.23)	0.69 (0.43-1.08)	0.91 (0.57-1.44)
Adjusted P value		0.34	0.11	0.68
**PTB**				
% (n/N)	6.3 (16/255)	5.2 (14/267)	10.5 (25/239)	14.2 (38/268)
OR (95% CI)	1	0.83 (0.40-1.73)	1.75 (0.91-3.36)	2.47 (1.34-4.55)
Adjusted OR (95% CI)	1	0.80 (0.38-1.71)	1.63 (0.79-3.35)	2.1 (1.01-4.37)
Adjusted P-value		0.56	0.19	0.04

The analyses were adjusted for the maternal age, BMI, duration of infertility, type of infertility(Primary/Secondary infertility), basal antral follicle count, fertilization method(IVF/ICSI), endometrial thickness on the trigger day and development stage of embryo(cleavage/blastocyst). OR, odds ratio; CI, confidence interval; SGA, small-for-gestational age; LBW, low birthweight; LGA, large-for-gestational age; PTB, preterm birth.

## Discussion

Among PCOS patients with a BMI<24 kg/m^2^, different AMH levels had no significant effect on the outcomes of singleton offspring, including PTB, SGA, LBW, macrosomia and LGA. However, among patients with a BMI ≥24 kg/m^2^, serum AMH>6.45 ng/ml was an independent risk factor for PTB but not for SGA, LBW, macrosomia or LGA.

### Comparison With Current Studies

Currently, regardless of whether PCOS patients achieve pregnancy naturally or through IVF, the risks of perinatal complications, including pregnancy-induced hypertension, gestational diabetes mellitus, miscarriage and PTB, may increase (7, 25). However, no clear indicators exist concerning the exact risk factors for adverse perinatal outcomes in the PCOS population. For a long time, the serum AMH levels have been considered an indicator of ovarian reserve, and an increase in AMH levels may suggest the existence of PCOS (26, 27). Additionally, the AMH level in the PCOS population is significantly higher than that in the non-PCOS population (13, 15, 28). To our best knowledge, only three clinical studies have analyzed the relationship between the serum AMH levels and preterm birth to date. J.Y. Hsu et al. (10) performed a retrospective cohort study to investigate the association between the AMH levels and risk of adverse obstetric outcomes. Among women with PCOS, those who delivered prematurely had substantially higher AMH levels (18 vs. 6.4 ng/mL; P=0.003) than those who delivered at term. Additionally, all women with PCOS and AMH levels above the 90th percentile had premature deliveries. Another retrospective cohort study performed in China including 25,165 IVF cycles showed that preterm deliveries were predominant among PCOS patients with AMH levels above the 75th percentile (9.75 ng/mL) (adjusted P<0.0001; AOR=4.0; 95% CI= 1.94-8.08)) and patients with frozen-thawed embryo transfer (FET) with AMH levels higher than the 90th percentile (10.10 ng/mL) (adjusted P<0.05; AOR=2.0; 95% CI=1.16-3.36) (9). Recently, a secondary analysis of data from two multicenter randomized clinical trials including a non-IVF PCOS population indicated that 62% of participants who delivered preterm had AMH levels above the 75th percentile (>9.3 ng/ml) (8). However, the article did not analyze different BMI groups, particularly overweight PCOS patients who may be more severely ill (19, 21). Several studies have explored the impact of the maternal BMI on the safety indicators for offspring, such as the PTB rate. A meta-analysis showed that different maternal BMIs have different effects on the incidence of PTB in offspring (29). Another Chinese cohort study showed that women who are overweight, obese, and nulliparous are at an increased risk of elective preterm birth (overweight women: OR = 1.36, 95% CI: 1.18–1.56; obese women: OR = 2.94, 95% CI: 2.04–4.25) (30). However, in a retrospective cohort study of 1680 women with PCOS undergoing FET, women who were overweight or obese did not exhibit significant differences in the rate of PTB compared with women of normal weight (31). The influence of the mother’s BMI on the PTB rate of children was also present in the PCOS population, and it was more significant in the high AMH group. In this study, among PCOS patients with a BMI<24 kg/m^2^, no significant correlation was found between elevated AMH levels and PTB or neonatal outcomes. However, among PCOS patients with a BMI>24 kg/m^2^, the PTB rate of singleton offspring was significantly increased when the AMH level was greater than the 75th percentile (6.45 ng/ml).

### Possible Biological Mechanism

Our study showed that elevated AMH levels are an independent risk factor for PTB among singletons of overweight PCOS patients, but the exact biological mechanism remains unclear. However, a direct link may exist between the AMH level and PTB, a finding that is biologically reasonable. The serum AMH level of PCOS patients is higher than that of non-PCOS individuals, indicating that AMH is an important indicator that can be used to assist in the diagnosis of PCOS (27). This dynamic does not occur only in the nonpregnancy period; the AMH level of the PCOS population during pregnancy is also significantly higher than that of the non-PCOS population, and the increase in the AMH level of PCOS patients may affect the endocrine system of the fetus and offspring (28). Research on its related mechanism is concentrated mainly on animal models. AMH is both a gonadal hormone and a putative paracrine regulator of neurons, the uterus, and the placenta, and high AMH levels can lead to miscarriage phenotypes in mouse model studies (32). We speculate that this phenomenon may be comparable to PTB in humans. Current studies have shown that AMH type II receptors exist on the surface of the myometrium and endometrial stroma and gland cells (33–35). Additionally, several studies have confirmed that AMH has a significant proapoptotic effect on tissues of Müllerian duct origin (36). Therefore, high AMH levels may inhibit myometrial hyperplasia during pregnancy, leading to uterine volume limitation and an increased risk of PTB (37). Alternatively, high AMH levels in early pregnancy can directly alter decidual and/or placental formation (37), as discussed by Hsu et al. (10). We also showed the relationship between high AMH levels and PTB. Interestingly, this effect was more significant in overweight PCOS patients, but this relationship was not significant in nonoverweight PCOS patients. Its specific biological mechanism warrants further study.

### Strengths and Limitations

To our best knowledge, only three clinical studies have analyzed the relationship between the serum AMH levels and premature delivery of singleton offspring (8–10). One concerns a non-IVF-assisted PCOS population (8), and the others describe IVF-assisted pregnancy populations (9, 10, 21). This study is the first clinical study to analyze the relationship between premature delivery and serum AMH in PCOS patients with different BMIs. It provides more data support for clinical consultation and new ideas for future clinical and basic research. This study also has certain limitations that must be addressed. First, this study was a retrospective cohort study with probable confounding factors. However, the sample size of this study was large, with a total of 2368 singleton births, and the influence of confounding factors was corrected through multiple logistic regression. Second, because of a lack of data, this study did not include insulin resistance in the grouping and analysis. However, we excluded gestational diabetes and hypertension to reduce the confounding effects of pregnancy complications on premature delivery and fetal development. Additionally, because of population differences and different monitoring machines, no uniform standard exists concerning how much AMH will affect the safety of offspring, and specific analyses must be performed based on the situation of their respective reproductive centers. This study was grouped according to the quartile of the serum AMH value. The results of the study showed that an AMH concentration above the 75th percentile (6.45 ng/ml) was an independent risk factor for singleton PTB in the overweight PCOS group. AMH is not a diagnostic indicator for PCOS patients, but high AMH is a characteristic of PCOS patients. Whether more effective indicators can represent the characteristics of PCOS patients warrants further study. Another limitation is the diagnosis of the PCOS population. Our diagnosis was primarily based on the Rotterdam criteria established in the 2003 Rotterdam consensus workshop (1). However, this study did not specifically distinguish between different PCOS phenotypes. Different PCOS phenotypes have differences in clinical and biological characteristics. Considering the diversity and heterogeneity of the clinical characteristics of PCOS patients, future clinical studies must also further analyze more specific phenotypic characteristics.

## Conclusion

In summary, among PCOS patients with a BMI<24 kg/m^2^, the AMH level had no significant effects on singleton offspring outcomes, such as PTB, SGA, LBW, macrosomia, and LGA. However, among PCOS patients with a BMI ≥24 kg/m^2^, serum AMH>6.45 ng/ml (75th percentile) is an independent risk factor for PTB but not an independent risk factor for SGA, LBW, macrosomia, or LGA. Therefore, for overweight PCOS patients with high AMH, obstetricians and pediatricians must focus on the pregnancy and perinatal period. However, the specific high AMH value must also be combined with the data of the respective reproductive centers, and larger, prospective, and well-designed randomized controlled studies are required for further analysis and research.

## Data Availability Statement

The raw data supporting the conclusions of this article will be made available by the authors, without undue reservation.

## Ethics Statement

The studies involving human participants were reviewed and approved by The Third Affiliated Hospital of Zhengzhou University. Written informed consent for participation was not required for this study in accordance with the national legislation and the institutional requirements.

## Author Contributions

GC designed the study. DZ and ZW selected the population to be included and excluded. DZ and ZW extracted and analyzed the data. YN reviewed the data. DZ and ZW drafted the article. All authors contributed to the article and approved the submitted version.

## Conflict of Interest

The authors declare that the research was conducted in the absence of any commercial or financial relationships that could be construed as a potential conflict of interest.

## Publisher’s Note

All claims expressed in this article are solely those of the authors and do not necessarily represent those of their affiliated organizations, or those of the publisher, the editors and the reviewers. Any product that may be evaluated in this article, or claim that may be made by its manufacturer, is not guaranteed or endorsed by the publisher.
